# Activation and Functional Alteration of Mucosal-Associated Invariant T Cells in Adult Patients With Community-Acquired Pneumonia

**DOI:** 10.3389/fimmu.2021.788406

**Published:** 2021-12-21

**Authors:** Lichen Ouyang, Mi Wu, Zhijun Shen, Xue Cheng, Wei Wang, Lang Jiang, Juan Zhao, Yeli Gong, Zhihui Liang, Xiufang Weng, Muqing Yu, Xiongwen Wu

**Affiliations:** ^1^ Department of Immunology, School of Basic Medicine, Tongji Medical College, Huazhong University of Science and Technology, Wuhan, China; ^2^ Department of Immunology, School of Medicine, Jianghan University, Wuhan, China; ^3^ Department of Respiratory and Critical Care Medicine, Tongji Hospital of Tongji Medical College, Huazhong University of Science and Technology, Wuhan, China

**Keywords:** community-acquired pneumonia (CAP), mucosal-associated invariant T cells (MAIT), bronchoalveolar lavage fluid (BALF), high-sensitivity C-reactive protein (hsCRP), immunopathogenesis

## Abstract

Community-acquired pneumonia (CAP) remains the significant infectious cause of morbidity and mortality worldwide. Although mucosal-associated invariant T cells (MAIT) play roles in the pathogenesis of children CAP and ICU-associated pneumonia, their roles in adult CAP are largely unexplored. In this study, we investigated the frequency, phenotype, and function of MAIT cells in peripheral blood and bronchoalveolar lavage fluid (BALF) of adult CAP patients. Our data indicate that MAIT-cell frequency is profoundly lower in the peripheral blood of CAP patients compared to that in healthy individuals. Furthermore, the circulatory MAIT cells express higher levels of CD69 and PD-1 compared to those in healthy individuals. In BALF of CAP patients, MAIT-cell frequency is higher and MAIT cells express higher levels of CD69 and PD-1 compared to their matched blood counterparts. Levels of IL-17A and IFN-γ are increased in BALF of CAP patients compared to those in BALF of patients with pulmonary small nodules. The IL-17A/IFN-γ ratio is significantly positively correlated with MAIT frequency in BALF of CAP patients, suggesting a pathogenic role of MAIT-17 cells in CAP. Of note, blood MAIT-cell frequency in CAP patients is strongly negatively correlated with high-sensitivity C-reactive protein (hsCRP) and neutrophil count percentage in blood. The ability of circulating MAIT cells in CAP patients to produce IFN-γ is significantly impaired compared to those in healthy individuals. In summary, our findings suggest the possible involvement of MAIT cells in the immunopathogenesis of adult CAP.

## Introduction

Community-acquired pneumonia (CAP) is an important infectious cause of mortality and morbidity worldwide which is caused by a wide spectrum of microbial pathogens outside a healthcare setting including bacteria, viruses, and fungi (1). The clinical manifestation of CAP can vary from mild pneumonia characterized by fever and cough to severe pneumonia characterized by acute respiratory distress syndrome, sepsis, and multiorgan dysfunction (2). The host innate and adaptive immune responses play pivotal roles in host defense against pathogens and in determining disease severity. Previous studies have demonstrated that dysfunctional neutrophils and CD4^+^Th17 cells are involved in the pathogenesis of CAP (3, 4). Recent studies suggest that unconventional T cells are equivalently important in the pathogenesis of CAP (5–7). Unconventional innate-like T cells, including invariant NKT cells, γδT cells, and mucosal-associated invariant T (MAIT) cells, are known to bridge the innate and adaptive arms of the immune response and play key roles in maintaining pulmonary homeostasis and the integrity of the lung epithelium (8).

MAIT cells are abundant in the liver as well as mucosal sites including the lung and gut, constituting 1%-10% of human peripheral blood T cells (9). They express a semi-invariant TCRVα7.2-Jα33/20/12 in humans and recognize derivatives of riboflavin metabolism in bacteria and yeasts presented on major histocompatibility complex class Ib-like molecules (MR1) (10). In addition, MAIT cells could be activated by cytokines such as IL-12 and IL-18 in a TCR-independent fashion which is important in the immune response against non-riboflavin-synthesizing bacteria and viruses (11). Upon activation, MAIT cells rapidly produce inflammatory cytokines (TNF-α, IFN-γ, and IL-17) and cytolytic molecules (granzyme B and perforin) (12). Studies from mouse studies suggest that MAIT cells play a protective role in both bacterial and viral pulmonary infection, such as Legionella longbeachae and influenza A (9, 13). In patients with chronic HBV infection and children with active pulmonary tuberculosis, MAIT frequency in the blood has been shown to decrease (14, 15). In addition, MAIT abundance was higher in the sputum of CAP patients compared to healthy controls determined by quantitative polymerase chain reaction and associated with disease severity (16). Moreover, tissue-resident MAIT cells producing IL-17 are increased in the bronchoalveolar lavage fluid (BALF) which has been suggested to contribute to IL-17-mediated inflammation in children with CAP (17). However, the role of MAIT cells in immunopathogenesis of adult CAP is largely unclear and yet to be unraveled. In this study, we aim to define the frequency, phenotype, function, and clinical relevance of MAIT cells in peripheral blood as well as BALF of adult CAP patients.

## Materials and Methods

### Patients and Ethical Statement

Eighteen adult CAP patients from Tongji Hospital (Wuhan, China) were enrolled in this study. The diagnosis of CAP was based on clinical presentation and chest radiograph. Patients with pulmonary tuberculosis, pulmonary cancer, asthma, and chronic obstructive pulmonary disease were excluded. Two groups of controls were included in this study, one of which consisted of 15 patients diagnosed with connective tissue diseases associated interstitial lung disease (CTD-ILD) and the other group consisted of 18 healthy donors. The diagnosis of CTD was based on clinical and serologic criteria according to the most recent European League Against Rheumatism (EULAR) recommendations. The diagnosis of ILD was made on the basis of high-resolution CT (HRCT) findings (18). To detect cytokines and define MAIT cells in BALF obtained from healthy respiratory mucosa, 6 patients with pulmonary small nodules (PSN) less than 5 mm in diameter who underwent electronic bronchoscopy for investigation were recruited. Patients with malignant lung nodules were excluded. The clinical parameters in blood which consisted of leukocyte number, percentage of the relative numbers of neutrophils in total leukocytes (neutrophil count percentage), percentage of the relative numbers of lymphocytes in leukocytes (lymphocyte count percentage), hypersensitive-CRP (hs-CRP), and erythrocyte sedimentation rate (ESR) were obtained from patients of CAP and CTD-ILD. Patient characteristics are shown in **Table 1**. The studies were reviewed and approved by the ethics committee of Tongji Medical College, Huazhong University of Science and Technology, and informed consents were obtained from all subjects.

**Table 1:** Patient Characteristics.

	HD	CAP	CTD-ILD	PSN	p value
Total, N	18	18	15	6	
Age (year), mean ± SD	39.2 ± 9.6	49.7 ± 17.6	56 ± 11.2	47.5 ± 12.4	0.0082
Male/female, N	10/8	11/7	5/10	3/3	0.43
Leukocytes (×10^9^/liter), mean ± SD		8.5 ± 4	7 ± 2.7	5.9 ± 1.8	0.29
Percentage of neutrophils in leukocytes (%)		71.3 ± 8.9	60.3 ± 11.1	56.5 ± 7	0.0019
Percentage of lymphocytes in leukocytes, (%)		20.1 ± 7.7	28.7 ± 9.1	32.6 ± 7.2	0.0047
ESR (mm/h), mean ± SD		43.5 ± 23.5	24.9 ± 19.8		0.017
hs-CRP (mg/L), mean ± SD		84.8 ± 87.6	8.3 ± 13.3		0.0054

HD, healthy donors; CAP, community-acquired pneumonia; CTD-ILD, connective tissue diseases associated interstitial lung disease; PSN, pulmonary small nodules; ESR, erythrocyte sedimentation rate; hs-CRP, high-sensitive C-reactive protein.

### Sample Collection

Bronchoscopy was performed with an electronic bronchoscope under topical lidocaine. For CAP or CTD-ILD patients, BALF (bronchoalveolar lavage fluid) was performed by instilling 50–70 ml of sterile 0.9% NaCl to the affected area identified radiologically and/or endoscopically. To obtain healthy BAL fluid, BALF was performed on the opposite side of the lung nodules in patients with pulmonary small nodules less than 5 mm in diameter. The BAL recovered was filtered through a 70-μm cell strainer and centrifuged for 10 min at 300g at 4°C. The supernatant was collected and stored at -80°C for subsequent cytokine detection. The cell pellets were resuspended in 1 ml of RPMI 1640 supplemented with 10% fetal bovine serum at 4°C for subsequent surface staining.

Peripheral venous blood samples were collected in heparinized tubes on the same day of bronchoscopy. Peripheral blood mononuclear cells (PBMC) were isolated by Ficoll density gradient centrifugation. PBMC were washed with phosphate-buffered solution (PBS) once and resuspended in RPMI 1640 supplemented with 10% fetal bovine serum. A portion of the cell pellets was used for subsequent surface staining at 4°C. The remaining cells were used for intracellular cytokine staining.

### Flow Cytometry

PBMC and BALF samples were incubated with Fc-receptor blocking reagent (BD Biosciences) at room temperature for 15 min and surface stained with the following fluorochrome-conjugated antibodies at 4°C for 40 min: CD45-APC (HI30, BioLegend), CD3-APC-cy7 (UCHT1, BioLegend), CD8-BV510 (SK1, BioLegend), CD4-BV421 (RPA-T4, BD Biosciences), CD161-PE (HP-3G10, BioLegend), CD161-BV421 (HP-3G10, BioLegend), Vα7.2-PE-cy7 (3C10, BioLegend), CD69-FITC (FN50, BioLegend), CD69-PE (FN50, BioLegend), and PD-1-BV421 (EH12.1, BD Biosciences). MAIT cells were identified as CD3^+^CD161^high^ Vα7.2^+^ cells.

For intracellular cytokine staining, PBMCs were cultured in RPMI 1640 supplemented with 10% FBS in the presence of phorbol 12-myristate 13-acetate (PMA) (25 ng/ml) and ionomycin (500 ng/ml) for 40 min followed by a 3.5-h incubation with brefeldin A in a 5% CO_2_ incubator at 37°C. IL-17A- and/or IFN-γ-producing cells were identified by intracellular staining with anti-IL-17A-FITC (BL168, BioLegend) and anti-IFN-γ-FITC (4S.B3, BioLegend) for 45 min after surface staining and fixation/permeabilization. Fluorescence Minus One (FMO) was used as negative control; the gating strategy for surface markers and intracellular cytokines is shown in **Supplemental Figure 1**. Cells were acquired by flow cytometry using a FACSVerse (BD Biosciences) and analyzed with FlowJo software (TreeStar).

### Cytokine Detection

Cytokines in BALF supernatant were determined by “LEGENDplex™ Human Inflammation Panel 1 (13 plex)” (BioLegend) with bead-based multiplex immunoassay according to the manufacturer’s instructions. The lower limit of detection for each cytokine is as follows: IL-1β: 1.5 pg/ml; IFN-α2: 2.1 pg/ml; IFN-γ: 1.3 pg/ml; TNF-α: 0.9 pg/ml; MCP-1: 1.1 pg/ml; IL-6: 1.5 pg/ml; IL-8: 2.0 pg/ml; IL-10: 2.0 pg/ml; IL-12p70: 2.0 pg/ml; IL-17A: 0.5 pg/ml; IL-18: 1.3 pg/ml; IL-23: 1.8 pg/ml; IL-33: 4.4 pg/ml. Data were collected using FACSVerse (BD Biosciences) and analyzed with FlowJo software (TreeStar).

### Statistics

Statistical analysis was performed using GraphPad Prism version 7 (GraphPad Software). Normally distributed data were analyzed by Student’s t test or ANOVA to assess differences between groups. For non-normally distributed data, the Mann–Whitney U, Kruskal–Wallis, and Wilcoxon signed-rank tests were utilized. p values for multiple comparisons were adjusted by the Benjamini–Hochberg method. Correlation analysis was evaluated with Spearman rank correlation. A p value of less than 0.05 was considered statistically significant. * for p< 0.05, ** for p<0.01, *** for p<0.001, **** for p<0.0001.

## Results

### MAIT Cells in the Peripheral Blood of CAP Patients Exhibited Increased Expression of Activation and Exhaustion Markers, Accompanied by a Decrease of Their Frequency

**Figure 1:**
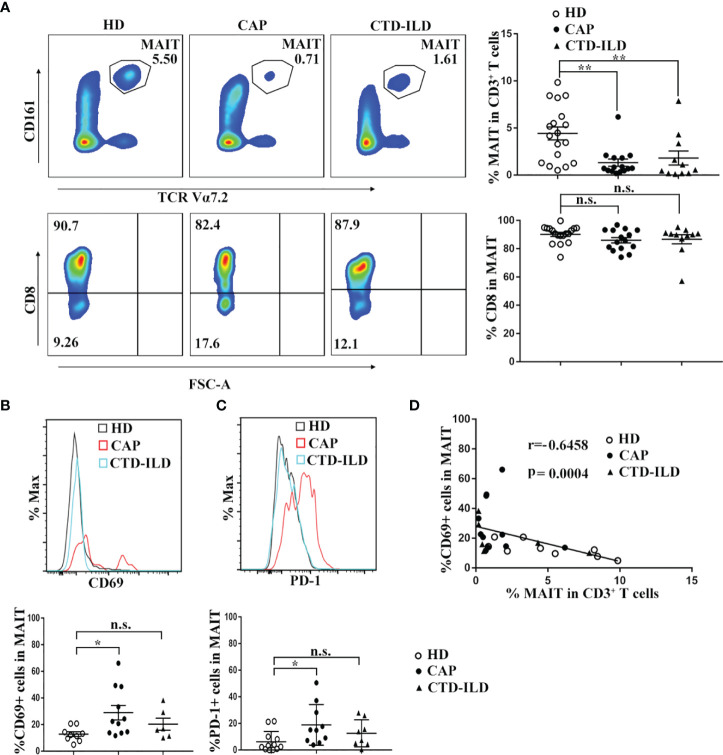
Frequency and phenotype of MAIT cells in peripheral blood of CAP patients. **(A)** Analysis of MAIT cells in the peripheral blood of HD (n = 18), CAP (n = 15), and CTD-ILD (n = 11) patients by flow cytometry. Representative plots of MAIT-cell frequency among CD3^+^ T cells and the proportion of CD8^+^ subset in MAIT cells of the three groups are shown in the left panel. Individuals and mean ± SEM are shown in the right panel. **(B)** Analysis of CD69 expression on MAIT cells in the peripheral blood of HD (n = 9), CAP (n = 11), and CTD-ILD (n = 6) patients by flow cytometry. Representative staining of CD69 and statistical dot plots of percentage of CD69+ MAIT of three groups are shown. **(C)** Analysis of PD-1 expression on MAIT cells in the peripheral blood of HD (n = 12), CAP (n = 10), and CTD-ILD (n = 8) patients by flow cytometry. Representative staining of PD-1 and statistical dot plots of percentage of PD-1+ MAIT of three groups are shown. **(D)** Spearman correlation of percentage of CD69+ MAIT cells with blood MAIT-cell frequency in HD (n = 9), CAP (n = 11), and CTD-ILD (n = 6) patients. HD, healthy donors; CAP, community-associated pneumonia; CTD-ILD, connective tissue diseases associated interstitial lung disease *P < 0.05, **P < 0.01, n.s., not significant.

Flow cytometry was applied to evaluate the frequency and phenotype of MAIT cells in CAP patients in comparison to healthy individuals. In parallel, we also defined MAIT frequency and phenotype in CTD-ILD patients which served as another control group. MAIT cells were identified as CD3^+^Vα7.2^+^ CD161^high^ T cells. Compared to healthy individuals, MAIT-cell frequencies among CD3^+^ T cells were significantly reduced in peripheral blood of CAP patients (p < 0.01, **Figure 1A**). Interestingly, we also found a profound decline of MAIT cells in the blood of CTD-ILD in comparison to healthy individuals (p < 0.01, **Figure 1A**). No significant difference in the proportion of the CD8^+^ subset within MAIT cells was found among the three groups.

Next, we investigated the expression of T-cell activation marker CD69 and exhaustion marker PD-1 on MAIT cells of three groups. In CAP patients, the percentages of CD69+ and PD-1+ MAIT cells were significantly higher than those in healthy individuals (p < 0.05 and p < 0.05, respectively. **Figures 1B, C** **)**. In contrast, there was no significant difference in the percentages of CD69+ and PD-1+ MAIT cells between healthy individuals and CTD-ILD patients. Remarkably, the percentage of CD69+ MAIT cells was negatively correlated with MAIT-cell frequency in peripheral blood (r = -0.6458, p = 0.0004, **Figure 1D**). Taken together, these results indicate that MAIT cells are reduced in frequency and activated in the peripheral blood of CAP patients.

### MAIT Cells Expressed Higher Levels of Activation and Exhaustion Markers in BALF Compared to Their Peripheral Counterparts in CAP Patients

**Figure 2:**
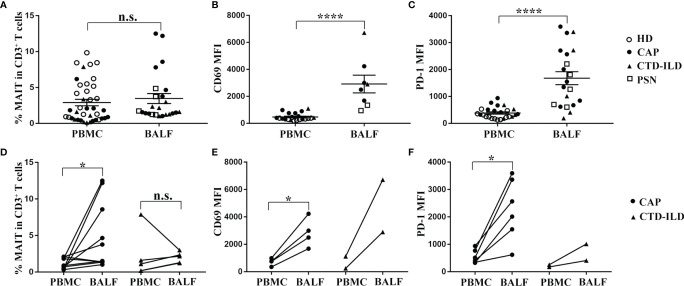
Comparison analysis of MAIT-cell frequency and phenotype between BALF and PBMC. **(A–C)** MAIT cells in BALF expressed higher levels of CD69 and PD-1 in comparison to those in peripheral blood. **(A)** Proportion of MAIT cells among CD3^+^ T cells in BALF of PSN (n = 5), CAP (n = 12), and CTD-ILD (n = 7) patients. **(B)** MFI of CD69 expression on MAIT cells in BALF of PSN (n = 2), CAP (n = 4), and CTD-ILD (n = 2) patients. **(C)** MFI of PD-1 expression on MAIT cells in BALF of PSN (n = 5), CAP (n = 10), and CTD-ILD (n = 5) patients. **(D–F)** MAIT cells were more enriched and exhibited a more activated and exhaustion phenotype in BALF compared to matched peripheral blood MAIT cells in CAP patients. **(D)** Paired analysis of MAIT-cell frequency in PBMC and BALF of CAP (n = 10) and CTD-ILD patients (n = 4). **(E)** Paired analysis of CD69 MFI on MAIT cells in PBMC and BALF of CAP (n = 4, respectively). **(F)** Paired analysis of PD-1 MFI on MAIT cells in PBMC and BALF of CAP (n = 6, respectively). *P < 0.05, ****P < 0.0001, n.s., not significant.

To determine whether MAIT cells are differently represented in the airway compared to the peripheral blood, we collected BALF samples in patients of CAP and CTD-ILD and detected the frequency as well as the phenotype of MAIT cells. Due to ethical reasons, the bronchoalveolar lavages could not be accessible in healthy individuals. Therefore, we obtained healthy BALF from the opposite side of the lung nodules in patients with pulmonary small nodules (PSN) less than 5 mm in diameter. We observed that MAIT-cell frequency in BALF was similar to that in the peripheral blood albeit with a slightly increasing trend (**Figure 2A**). In line with previous reports (17, 19) on MAIT-cell activation and exhaustion status in BALF and the peripheral blood, we also found that MAIT cells in BALF expressed higher levels of activation and exhaustion markers in comparison to those in the peripheral blood (p < 0.0001 and p < 0.0001, respectively, **Figures 2B, C** **)**.

Next, we further compared the frequency and phenotype of MAIT cells between BALF and their peripheral counterparts in CAP patients. MAIT cells were more abundant in BALF than in the peripheral blood from CAP patients (p < 0.05, **Figure 2D**). Moreover, CD69 and PD-1 expressions on MAIT cells were significantly higher in BALF than in the matched peripheral blood of CAP patients (p < 0.05 and p < 0.05, respectively, **Figures 2E, F** **)**. Altogether, we observed that MAIT cells were more enriched and exhibited a higher expression of activation and exhaustion markers in BALF compared to matched peripheral blood MAIT cells in CAP patients.

### Altered Function of MAIT Cells in Peripheral Blood of CAP Patients

**Figure 3:**
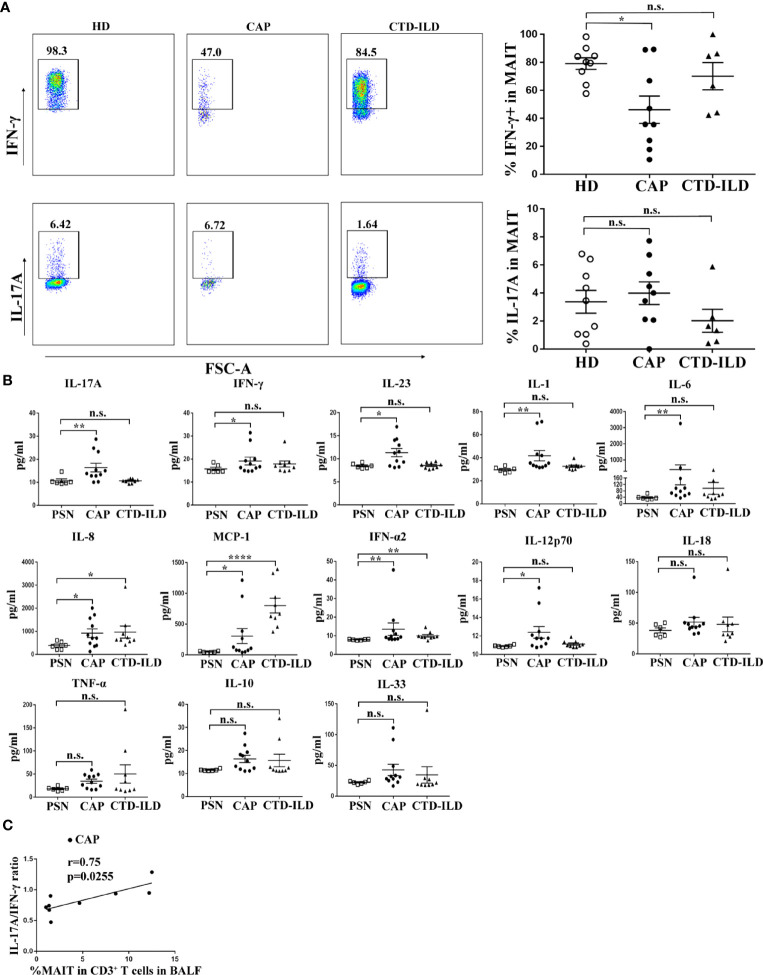
Functional evaluation of MAIT cells in PBMC and BALF. **(A)** Intracellular staining for IFN-γ and IL-17A of PBMC stimulated by PMA and ionomycin in HD (n = 9), CAP (n = 9), and CTD-ILD patients (n = 6). **(B)** Levels of cytokines in BALF of PSN (n = 6), CAP (n = 11), and CTD-ILD patients (n = 9) as determined by bead-based multiplex immunoassay are shown. **(C)** Spearman correlation of MAIT-cell frequency in BALF with a ratio of IL-17A/IFN-γ level in BALF in CAP (n = 9) patients. PSN, pulmonary small nodules. *P < 0.05, **P < 0.01, ****P < 0.0001, n.s., not significant.

MAIT cell function in peripheral blood was assessed by intracellular flow cytometry staining after stimulation with PMA and ionomycin. The proportion of IFN-γ+ cells was lower in MAIT cells of CAP patients compared to healthy individuals (p < 0.05, **Figure 3A**). In contrast, there was no significant difference in the proportion of IFN-γ+ cells in MAIT cells between CTD-ILD patients and healthy individuals (**Figure 3A**). There was no significant difference in the proportion of IL-17A+ MAIT cells in CAP patients and CTD-ILD patients compared to healthy individuals (**Figure 3A**).

Due to the insufficient cell number from bronchoscopy samples, it was not possible to directly evaluate cytokine produced by MAIT cells in BALF by intracellular flow cytometry staining. Then, we detected cytokines in the supernatants of BALF. Compared to healthy mucosa in patients of PSN, we found that the IL-17A and IFN-γ levels were significantly higher in BALF of CAP patients (p < 0.01 and p < 0.05, respectively, **Figure 3B**). IL-23, an IL-17-related cytokine, was also elevated in BALF of CAP patients (p < 0.05, **Figure 3B**). A significant positive correlation was found between the IL-17A/IFN-γ ratio and MAIT-cell frequency in BALF of CAP patients (r = 0.75, p = 0.02553, **Figure 3C**). Besides cytokines related to adaptive immunity, several innate inflammatory cytokines (IL-1, IL-6, IL-8, MCP-1, IFN-α2, and IL-12p70) were also found elevated in BALF of CAP patients, mirroring the local hyper-inflammatory environment. Collectively, these results indicate a reduced capacity of blood MAIT-cells to produce IFN-γ and a more pronounced hyper-inflammatory environment in BALF of CAP patients.

### MAIT-Cell Frequency in Blood Is Correlated With Clinical Parameters in CAP Patients

**Figure 4:**
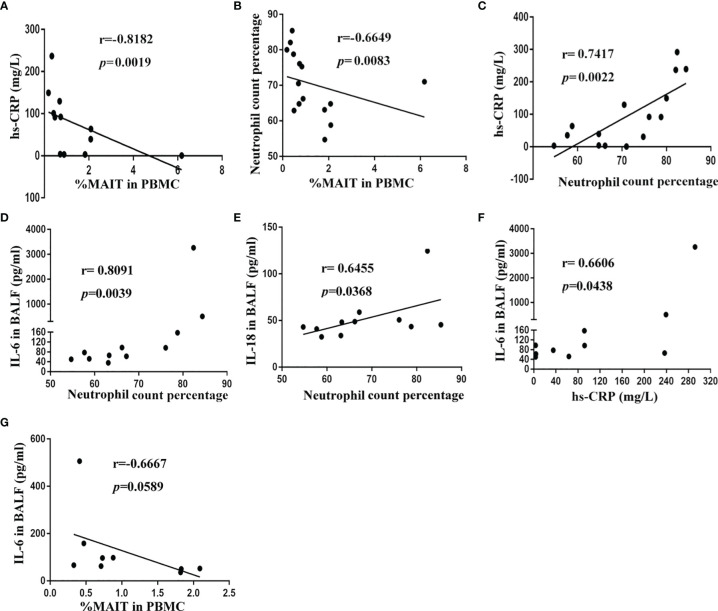
Correlation of MAIT-cell frequency and cytokine levels in BALF with clinical parameters in CAP patients. **(A)** Spearman correlation of blood MAIT-cell frequency with hs-CRP in blood (n = 12). **(B)** Spearman correlation of blood MAIT-cell frequency with neutrophil count percentage in blood (n = 15). **(C)** Spearman correlation of hs-CRP level with neutrophil count percentage in blood (n = 15). **(D, E)** Spearman correlation of IL-6 and IL-18 levels in BALF with neutrophil count percentage in blood (n = 11). **(F)** Spearman correlation of hs-CRP level with IL-6 level in BALF (n = 10). **(G)** Spearman correlation of blood MAIT-cell frequency with IL-6 level in BALF (n = 9).

To gain insight into the potential association of MAIT cells and pneumonia disease severity, we investigated correlations between MAIT-cell frequency and clinical variables. Results revealed that high-sensitivity C-reactive protein (hs-CRP) level and neutrophil count percentage strongly negatively correlated with circulating MAIT-cell frequency (r = -0.8182, p = 0.0019, and r = -0.6649, p = 0.0083, respectively, **Figures 4A, B** **)**. Unsurprisingly, high-sensitivity C-reactive protein (hs-CRP) level positively correlated with neutrophil count percentage in peripheral blood (r = 0.7417, p = 0.0022, **Figure 4C**). However, we did not find a significant correlation between MAIT-cell frequency in BALF and the above clinical variables (data not shown). Then, we further interrogated potential links among cytokine levels in BALF, MAIT-cell frequency, and clinical variables. Of note, only IL-6 and IL-18 levels in BALF positively correlated with neutrophil count percentage (r = 0.8091, p = 0.0039 and r = 0.6455, p = 0.0368, respectively, **Figures 4D, E** **)**. As expected, the IL-6 level in BALF positively correlated with the high-sensitivity C-reactive protein (hs-CRP) level (r = 0.6606, p = 0.0438, **Figure 4F**) and negatively correlated with circulating MAIT-cell frequency (r = -0.6667, p = 0.0589, **Figure 4G**).

## Discussion

Our study reveals profound MAIT-cell alterations in frequency, phenotype, and function in patients with CAP. The frequency of MAIT-cells was lower in the blood compared to healthy donors, while MAIT frequency in BALF was higher compared to their PBMC counterparts in CAP patients. The decline in blood MAIT-cell frequency was associated with high activation. Of note, the function of blood MAIT cells was impaired as revealed by decreased IFN-γ production. Finally, blood MAIT-cell frequency negatively correlated with the hs-CRP level and neutrophil count percentage in blood and IL-6 levels in BALF.

The reduction in blood MAIT-cell frequency in patients of CAP in the current study is consistent with previous studies in patients with active tuberculosis and sepsis as well as recent studies in patients with COVID-19 (15, 20, 21). The dramatic drop in blood MAIT-cell frequency may have several reasons. Firstly, in line with other studies (22, 23), our results showed that MAIT cells were enriched in the BALF compared to matched blood MAIT cells in CAP patients. MAIT cells constitutively express high levels of chemokine receptors CCR5, CCR6, and CXCR6 which have a propensity to promote migration to the inflamed tissues (24). Thus, it is reasonable to speculate that decreased circulating MAIT cells in CAP patients result from their migration into the inflamed lung. Secondly, expressions of activation marker CD69 and exhaustion marker PD-1 on blood MAIT cells of CAP patients were significantly higher compared to those of healthy controls. Furthermore, MAIT-cell frequency was inversely correlated with CD69 expression, implying that activation-induced cell death may also contribute to MAIT-cell reduction. Another possible explanation which could not be excluded is TCR downregulation on MAIT cells in the face of chronic antigen or cytokine stimulation. In our study, blood MAIT-cell frequency in patients of CTD-ILD also declined which is consistent with studies in patients of autoimmune diseases including SLE and RA (25). The reduction of MAIT cells in peripheral blood may result from chronic inflammatory activity or the application of steroids or immunosuppressive drugs (19).

In this study, the activation and exhaustion marker of MAIT cells in CAP patients were significantly higher in BALF than in the matched peripheral blood. Similar results have been revealed in patients of sarcoidosis and COVID-19 and children with CAP (17, 22, 26). In these studies, the local intense inflammatory environment is supposed to contribute to activation and exhaustion of MAIT cells. Our results demonstrated that innate inflammatory cytokines (IL-1, IL-6, IL-8, MCP-1, IFN-α2, and IL-12p70) were significantly increased in BALF of CAP patients, suggesting that the local hyper-inflammatory environment may be partly responsible for the MAIT cell activation in CAP patients.

MAIT cells rapidly produce cytokines and cytotoxic molecules upon TCR or cytokine-mediated activation (12). Majority of circulating MAIT cells secrete IFN-γ and TNF-α after stimulation with PMA and ionomycin, while only a minority of them produce IL-17 (14). Functional impairment of blood MAIT cells with decreased IFN-γ and increased IL-17 has been reported in patients with COPD and COVID-19 (26, 27). In our study, the proportion of IFN-γ+ cells was lower in blood MAIT cells of CAP patients compared to healthy controls. This decrease in IFN-γ could be partly due to activation-induced MAIT cell hypo-responsiveness which is previously reported on NKT cells (28). Given that PD-1 expression on T cells is associated with low responsiveness of these cells (29), we speculate that the enhanced expression of PD-1 on blood MAIT cells of CAP patients may also contribute to the decrease of IFN-γ.

Mucosal MAIT cells are functionally distinct from their peripheral blood counterparts. Compared to blood MAIT cells, MAIT cells in mucosal tissues exhibit an IL-17-biased proinflammatory phenotype with less IFN-γ secretion (23). In children with CAP, the production of IL-17 by MAIT cells in BALF is significantly increased and MAIT-17 cells are the main contributor of elevated IL-17 levels in BALF (17). Inflammatory cytokines from CD14^+^ monocytes are supposed to lead to MAIT-17 differentiation which are responsible for IL-17-mediated inflammation (17). In our study, we did not perform intracellular cytokine staining due to the lack of sufficient cell counts; thus, we detected cytokines in BALF instead to imply the functional alteration of MAIT cells. Our results showed that IL-17 and its related cytokine IL-23 were significantly elevated in BALF of CAP patients compared to controls. A significant positive correlation was found between IL-17A/IFN-γ ratio and MAIT-cell frequency in BALF of CAP patients. Considering the elevated MAIT-cell frequency and the main contribution of IL-17 by MAIT cells in BALF, we speculate that MAIT cells in BALF of CAP patients in our study might display a MAIT-17-biased phenotype which plays a pivotal role in the pathogenesis of CAP.

Our study has several limitations. Firstly, the sample size is limited. Although our patients had a homogenous phenotype, larger studies are warranted to confirm our present results. Secondly, due to the insufficient cell number from bronchoscopy samples, it was not possible to directly evaluate cytokines produced by MAIT cells in BALF. Further studies using resected lung tissues are required to solve this issue. Lastly, bronchoalveolar lavages were not accessible in healthy individuals for ethical reasons, although our study included two control groups.

In summary, we describe a profound decline of blood MAIT cells and an enrichment of MAIT cells in the airways of CAP patients. Blood MAIT cells with functional impairment exhibited an increased expression of activation and exhaustion markers. Altogether, these findings provide new insights into the potential role of MAIT cells in the pathogenesis of CAP, which may serve as a potential biomarker and target for the evaluation and treatment of severe CAP.

## Data Availability Statement

The original contributions presented in the study are included in the article/**Supplementary Material**. Further inquiries can be directed to the corresponding authors.

## Ethics Statement

The studies involving human participants were reviewed and approved by Tongji Medical College, Huazhong University of Science and Technology. The patients/participants provided their written informed consent to participate in this study.

## Author Contributions

LO, MW, XFW, XWW, and MY conceived and designed the experiments. LO, MW, ZS, XC, LJ, JZ, WW, and YG performed the experiments. LO, MW, and MY collected the clinical samples. LO, MW, ZS, XC, WW, YG, ZL, XFW, XWW and MY analyzed the data. LO, XW, MY, and XWW prepared the manuscript. All authors critically reviewed the manuscript. All authors contributed to the article and approved the submitted version.

## Funding

This work was supported by the National Natural Science Foundation of China (81871235, 81871226) and the Scientific Research Foundation of Jianghan University.

## Conflict of Interest

The authors declare that the research was conducted in the absence of any commercial or financial relationships that could be construed as a potential conflict of interest.

## Publisher’s Note

All claims expressed in this article are solely those of the authors and do not necessarily represent those of their affiliated organizations, or those of the publisher, the editors and the reviewers. Any product that may be evaluated in this article, or claim that may be made by its manufacturer, is not guaranteed or endorsed by the publisher.
