# Supplementary figures and images for: Activation and Functional Alteration of Mucosal-Associated Invariant T Cells in Adult Patients With Community-Acquired Pneumonia
Source: Front Immunol. 2021 Dec 21;12:788406. doi: 10.3389/fimmu.2021.788406 (PMC8724213; doi:10.3389/fimmu.2021.788406)

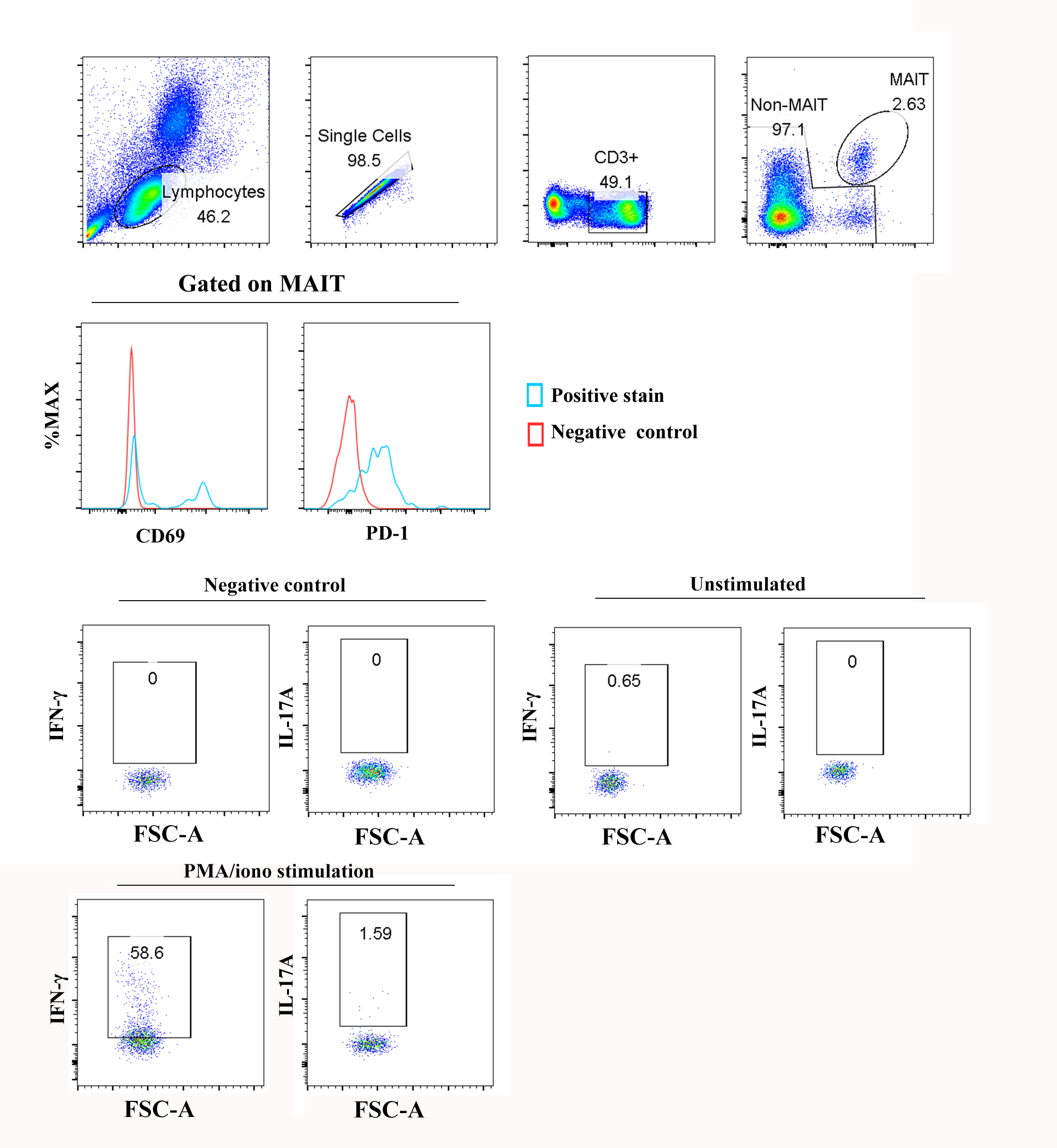

Supplement: Supplementary file 1 [file Image_1.tif]
